# CircIBTK inhibits DNA demethylation and activation of AKT signaling pathway via miR-29b in peripheral blood mononuclear cells in systemic lupus erythematosus

**DOI:** 10.1186/s13075-018-1618-8

**Published:** 2018-06-08

**Authors:** Xin Wang, Chengzhong Zhang, Zhouwei Wu, Yue Chen, Weimin Shi

**Affiliations:** 1 Department of Dermatology, Shanghai General Hospital, Shanghai Jiaotong University School of Medicine, Haining Road 100, Shanghai, 200080 China; 2 Department of Dermatology, Shanghai General Hospital of Nanjing Medical University, Haining Road 100, Shanghai, 200080 China

**Keywords:** Systemic lupus erythematosus, Circular RNAs, miR-29b, DNA methylation, AKT signaling

## Abstract

**Background:**

Systemic lupus erythematosus (SLE) is a chronic and incurable autoimmune disease involving the dysfunction of lymphocytes. Circular RNAs (circRNAs) are noncoding RNAs (ncRNAs) with a covalently closed loop structure, with abnormal expression in various human diseases may participate in the pathogenesis, while further study is needed in SLE. In this study, we aimed to find the circRNAs abnormally expressed in SLE and explore the function of circRNAs in SLE.

**Methods:**

CircRNA sequencing was used to find the abnormally expressed circRNA and qRT-PCR was used to detect the expression. Correlation analysis was used to analyze the correlation between circIBTK or miR-29b and clinicopathological variables in patients with SLE. Cell culture, nuclear-cytoplasmic fractionation, qRT-PCR, transfection, luciferase reporter assay, western blot analysis, DNA extraction and global methylation analysis were used to explain the function of circIBTK and miR-29b in the progression of SLE. SPSS 18.0 software was used to perform statistics.

**Results:**

We found that the expression of circIBTK was downregulated in SLE and correlated with Systemic Lupus Erythematosus Disease Activity Index (SLEDAI) score, anti-double-stranded (ds)DNA and complement C3 level in patients with SLE. Then miR-29b expression was upregulated in SLE and correlated with SLEDAI score, anti-dsDNA and complement C3 level in patients with SLE. Mechanistic investigations indicated that miR-29b could induce DNA demethylation and activate the AKT signaling pathway and circIBTK might reverse the DNA demethylation and activation of the AKT signaling pathway induced by miR-29b via binding to miR-29b in SLE.

**Conclusions:**

CircIBTK was downregulated in SLE and might regulate DNA demethylation and the AKT signaling pathway via binding to miR-29b in SLE. CircIBTK and miR-29 could also act as biomarkers and therapeutic targets for SLE.

**Electronic supplementary material:**

The online version of this article (10.1186/s13075-018-1618-8) contains supplementary material, which is available to authorized users.

## Background

Systemic lupus erythematosus (SLE) is a chronic and incurable autoimmune disease, which involves multiple organs, including skin, kidneys, and central nervous system [1, 2]. In recent years many studies have been dedicated to elucidating the pathogenesis of SLE, which is still unknown. A very significant pathophysiological feature of SLE is the dysfunction of T cells, B cells, and dendritic cells (DC) [3–6], and research on the dysfunction of immune cells through SLE progression is a hot topic.

Circular RNAs (circRNAs) are noncoding RNAs (ncRNAs) with a covalently closed loop structure without 5′ cap and a 3′ polyadenylated tail. They are highly stable and widely exist in eukaryotic cells. They do not usually encode protein, but can occur in any genomic region, regulating gene expression in eukaryotes. Recently circRNAs were found to be enriched with functional microRNA (miRNA) binding sites and could act as miRNA sponges to regulate gene expression [7, 8]. Furthermore, more and more studies have demonstrated that circRNAs are expressed abnormally in many human diseases and might play a significant role in the pathogenesis and diagnosis of these diseases [9–13].

DNA methylation is abnormal in T cells and B cells from patients with SLE. Besides global hypomethylation on lupus T and B cells, the gene-specific and site-specific methylation has been identified to be responsible for SLE. Aberrant DNA methylation plays an important role in the initiation and development of SLE and provides an insight into the related diagnosis biomarkers and therapeutic options in SLE [14, 15]. Some studies showed that the PI3K/AKT signaling pathway played a role in the differentiation of peripheral B cell and T cell homeostasis [16–19]. Many researchers also found there is abnormal activation of the PI3K/AKT signaling pathway in SLE and they thought this might participate in the pathogenesis of SLE. For example, in MRL-lpr lupus models, CD4+ T cells show higher levels of AKT activation than in wild-type mice [20]. Anomalous activity of AKT kinases has been documented in peripheral blood B cells and T cells from patients with SLE [21, 22]. Rapamycin has been successfully used to target the AKT/mTOR axis for treatment of patients with SLE [3].

In this study, we characterized a new circRNA, hsa_circ_0077179, which was derived from the IBTK gene locus, termed circIBTK, and which was downregulated in patients with SLE. We further estimated the value of circIBTK as a biomarker in SLE and studied the role of circIBTK in the DNA hypomethylation and abnormal activation of the AKT signaling pathway.

## Methods

### Patients and controls

This research was approved by the Institutional Research Ethics Committee of Shanghai General Hospital and abided by the ethical guidelines of the Declaration of Helsinki. Informed consent was obtained from all the patients involved in this study. The diagnosis of SLE was established based on the 1997 revised American Rheumatism Association criteria and all the patients were diagnosed with SLE for the first time or without treatment with glucocorticoid or immunosuppressive agents for one month. Clinical data used in this research on 42 patients and 35 age-matched and gender-matched healthy controls from Shanghai, China are summarized in Additional file 1: Table S1.

### Cell isolation and culture

Whole blood (10 ml) was collected in EDTA collection tubes from each subject, and human peripheral blood mononuclear cells (PBMCs) were isolated by density-gradient centrifugation using Ficoll-Paque Plus (GE Healthcare Biosciences) and cultured in Roswell Park Memorial Institute (RPMI) 1640 medium with 10% fetal bovine serum at 37 °C with 5% CO2 for 24 h before transfection.

### CircRNA sequencing analysis

Total RNA was extracted from PBMCs using Trizol (Invitrogen, Carlsbad, CA, USA) according to the instructions of the manufacturer. Before sequencing, RNAs were digested with Rnase R (Epicentre Technologies, Madison, WI, USA) to remove the linear RNAs and enrich the circular configuration. The sequencing analysis was performed on Illumina HiSeq2000 Platform (Illumina Inc., San Diego, CA, USA).

### Nuclear-cytoplasmic fractionation and quantitative real-time PCR (qRT-PCR)

Total RNA was extracted using the DNA/RNA Isolation Kit (DP422, Tiangen Biotech, Beijing, China) according to the instructions supplied by the manufacturer. The nuclear and cytoplasmic fractions were isolated using NE-PER Nuclear and Cytoplasmic Extraction Reagents (Thermo Scientific). Total RNA from the nuclear and cytoplasmic fractions was isolated with Trizol (Invitrogen) according to the instructions provided by the manufacturer. After reverse transcription, complementary DNA (cDNA) was amplified by using SYBR-Green Premix (Takara, Otsu, Japan). MiR-29b expression levels were detected using the Hairpin-itTM Quantitation PCR Kit (GenePharma, Shanghai, China). The expression of miR-29b and nuclear circIBTK were normalized to the expression of U6 and the expression of IBTK and cytoplasmic circIBTK were normalized to the expression of glyceraldehyde-3-phosphate dehydrogenase (GAPDH). The data were analyzed using the delta cycle threshold (Ct) method. Primers for circIBTK were designed by Genechem (Shanghai, China). The primers for IBTK were forward, 5’CTTACATGTCTGCTGCTTTTGG-3′; reverse, 5′- GAGACACATAAGCAATTCACTGC-3′. The primers for GAPDH were forward, 5′- GAAGATGGTGATGGGATTTC-3′; reverse, 5’-GAAGGTGAAGGTCGGAGT-3′. The primers for U6 were forward, 5’-TCGCTTCGGCAGCACATA-3′; reverse, 5’-TTTGCGTGTCATCCTTGC-3′.

### Transfection and luciferase reporter assay

MiR-29b mimics, miR-29b inhibitor and NC oligonucleotides were obtained from GenePharma. CircIBTK expression plasmids were designed by Genechem. SiRNA for circIBTK: sequence, 5’-GGAAUUUCCUUGUCAUAAAUG-3′, anti-sequence, 5’-UUUAUGACAAGGAAAUUCCUU-3′. Oligonucleotides were transfected by Hiperfect transfection reagent (Qiagen, Valencia, CA, USA) and plasmids were transfected by Lipofectamine 3000 (Invitrogen) into cells. All cells were incubated for 48 h afterwards. For circIBTK and miR-29b luciferase reporter assay, the circIBTK sequences containing wild-type miR-29b predicted binding sites were inserted into the region directly downstream of a cytomegalovirus (CMV) promoter-driven firefly luciferase cassette in a pCDNA3.1 vector. For PTEN 3’ UTR and miR-29b luciferase reporter assay, the PTEN 3’ UTR sequences containing two wild-type miR-29b predicted binding sites were inserted into the region directly downstream of a CMV promoter-driven firefly luciferase cassette in a pCDNA3.1 vector. Mutant reporter plasmids were prepared using Mutagenesis Kit (Stratagene, La Jolla, CA, USA). All constructs were verified by sequencing. PBMCs were seeded into 96-well plates and were co-transfected with a mixture of 30 ng of firefly luciferase reporter, 5 ng of pRL-TK Renilla luciferase reporter, and miRNA mimics or inhibitor. After 48 h of incubation, the firefly and Renilla luciferase activities were quantified using the Dual Luciferase Assay System (Promega, Madison, WI, USA).

### Western blot analysis

Western blot analysis was carried out using standard procedures. Cells were lysed using radioimmunoprecipitation (RIPA) lysis buffer (Boster, Wuhan, China). Protein concentrations were detected using bicinchoninic acid (BCA) Protein Assay Kit (Thermo Fisher Scientific, Rockford, IL, USA). Total proteins were separated by 10% sodium dodecyl sulfate polyacrylamide gel electrophoresis (SDS-PAGE) and transferred onto a polyvinylidene difluoride (PVDF) membrane (Millipore, USA). Antibodies used in the assays were β-actin (number 3700; Cell Signaling Technology, Danvers, MA, USA), AKT (pan) (number 4691; Cell Signaling Technology), phospho-AKT (Ser473) (number 4060; Cell Signaling Technology), PTEN (number 9552; Cell Signaling Technology).

### DNA extraction and global methylation analysis

Genomic DNA was extracted from PBMCs using the DNA/RNA Isolation Kit (Tiangen Biotech) according to the instructions provided by the manufacturer. Global DNA methylation status in DNA samples was detected using the Methylflash Methylated DNA Quantification Kit (Epigentek, Farmingdale, NY, USA) according to the manufacturer’s recommended protocol.

### Statistical analysis

Statistical analysis was performed using the SPSS program (version 18.0; SPSS, Chicago, IL, USA). The statistical significance of differences between two groups was tested using Student’s *t* test or the chi square (χ2) test. Multiple comparisons were performed using one-way analysis of variance (ANOVA) followed by the Newman–Keuls test. Spearman’s analysis was used to test correlation. *P* < 0.05 was considered as statistically significant.

## Results

### CircIBTK expression was downregulated in SLE and correlated with clinicopathological variables in patients with SLE

**Fig. 1:**
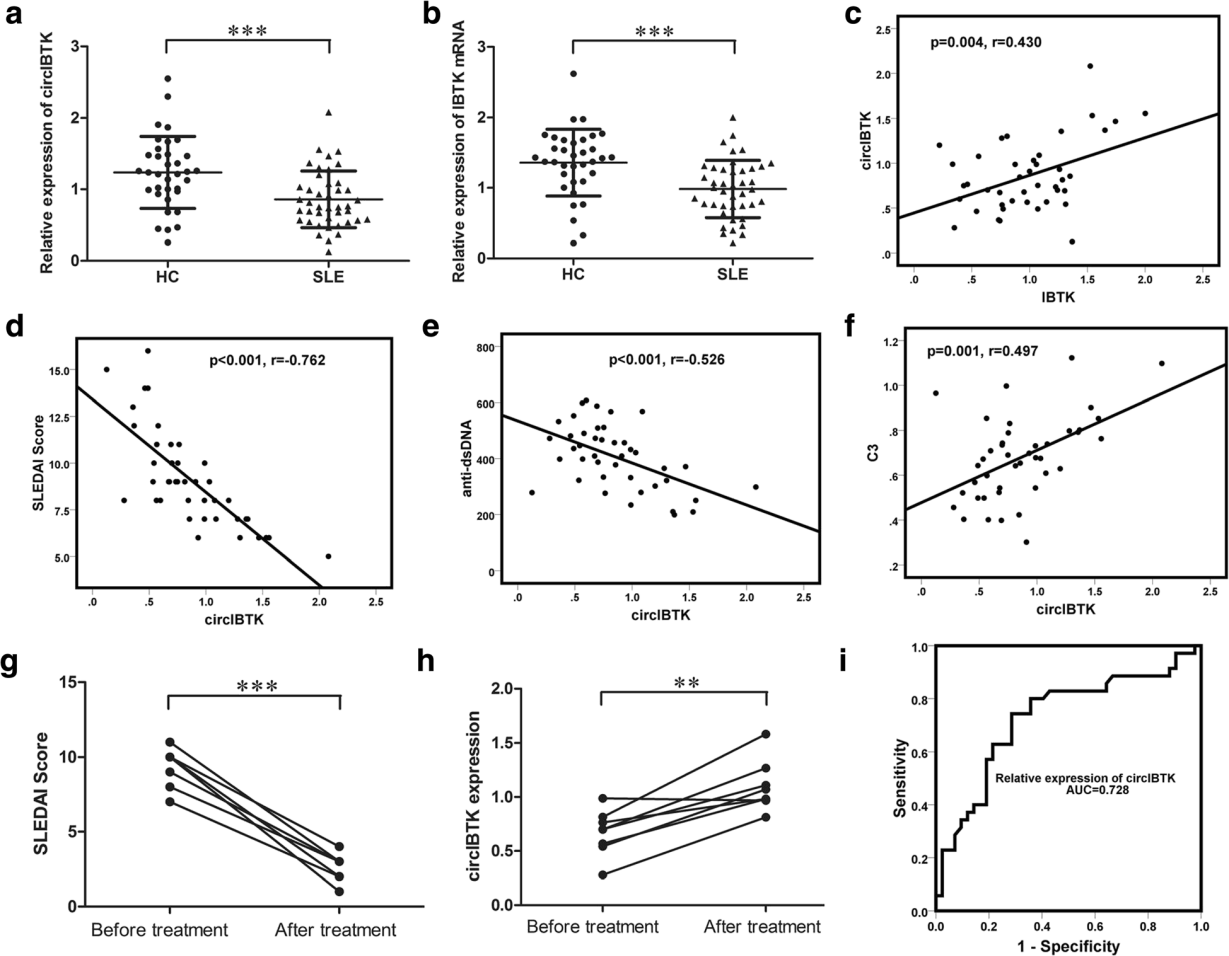
CircIBTK expression was downregulated in systemic lupus erythematosus (SLE) and correlated with clinicopathological variables in patients with SLE. **a**, **b** Expression of circIBTK and IBTK mRNA in periperhal blood mononuclear cells (PBMCs) from 42 patients with SLE and 35 healthy controls (HC) compared using the unpaired Student’s *t* test. **c** Correlation between expression of circIBTK and IBTK mRNA levels analyzed with Spearman’s analysis. **d**-**f** Correlation between expression of circIBTK and the Systemic Lupus Erythematosus Disease Activity Index (SLEDAI) score, anti-dsDNA titer or complement C3 level analyzed with Spearman’s analysis. **g**, **h** SLEDAI score and circIBTK expression in eight patients who achieved significant clinical improvement after systematic treatment; data were compared using the paired Student’s *t* test. The SLEDAI score in two patients declined from 11 to 3 and so there are two lines that overlap in **g**. **i** ROC curve of relative circIBTK expression for differentiating 42 patients with SLE from 35 HC. Results are represented as mean ± SD. **P < 0.01, ***P < 0.001

To screen the circRNA expression profiles in SLE, we first performed circRNA sequencing to find the differently expressed circRNAs in the peripheral blood mononuclear cells (PBMCs) of patients with SLE compared to healthy controls (HC) (Table 1). Hsa_circ_0077179, which was derived from the IBTK gene locus, termed circIBTK, was significantly downregulated in SLE and was chosen to study the function in SLE. Then, we verified the expression of circIBTK using qRT-PCR in PBMCs obtained from 42 patients with SLE and 35 healthy controls. Consistent with circular RNA sequencing data, the expression level of circIBTK in SLE was significantly lower than that in healthy controls (Fig. 1a). We also detected the expression of IBTK mRNA using qRT-PCR in PBMCs from those samples. The result showed that the expression of IBTK messenger RNA (mRNA) in SLE was also lower than that in HC (Fig. 1b). There was correlation between the expression of circIBTK and IBTK mRNA levels in SLE (Fig. 1c).

**Table 1:** Differently expressed circRNAs in PBMCs from patients with SLE compared to healthy controls

circRNA	Fold change	Up/down	Gene symbol
hsa_circ_0092285	26.175	up	PNKP
hsa_circ_0059802	14.367	up	DNMT3B
hsa_circ_0006095	10.128	up	ZNF148
hsa_circ_0009289	8.623	up	ATAD3A
hsa_circ_0001492	7.908	up	ERBB2
hsa_circ_0000267	6.461	up	FAM53B
hsa_circ_0009242	5.788	up	SDF4
hsa_circ_0001393	4.390	up	STX18
hsa_circ_0000615	4.457	up	ZNF609
hsa_circ_0006510	3.359	up	DYNC2H1
hsa_circ_0000141	2.920	up	SMG5
hsa_circ_0005188	2.519	up	DHX34
hsa_circ_0044234	0.521	down	CDC27
hsa_circ_0011018	0.420	down	ARID1A
hsa_circ_0006385	0.402	down	EIF3D
hsa_circ_0004033	0.323	down	ADPGK
hsa_circ_0001937	0.298	down	TRMT2B
hsa_circ_0000444	0.267	down	MED13L
hsa_circ_0008916	0.255	down	NUDT4
hsa_circ_0022058	0.198	down	NUP160
hsa_circ_0077179	0.117	down	IBTK
hsa_circ_0009035	0.109	down	RACGAP1
hsa_circ_0022599	0.091	down	ATL3
hsa_circ_0022812	0.052	down	POLA2
hsa_circ_0003060	0.040	down	SUCLG2

*circRNA* Circular RNA, *PBMC* peripheral blood mononuclear cell, *SLE* systemic lupus erythematosus

To explore whether circIBTK might be a potential biomarker in the clinical estimation of the activity of SLE, data on clinicopathological variables were collected in 42 patients with SLE (Additional file 1: Table S1) and correlation between these data and circIBTK was tested. There was strong inverse correlation between circIBTK expression and the Systemic Lupus Erythematosus Disease Activity Index SLEDAI score in patients with SLE. CircIBTK expression was also inversely correlated with anti-dsDNA titer and positively correlated with complement C3 level (Fig. 1d, e and f). Furthermore, we collected PBMCs from eight patients when they achieved significant clinical improvement after systematic treatment. CircIBTK expression was notably increased when these patients achieved significant clinical improvement (Fig. 1g and h). These results demonstrated that circIBTK could act as a biomarker to estimate the activity of SLE and verify the effectiveness of the treatment of SLE. To assess the diagnostic value of circIBTK for SLE, we performed receiver operating characteristic (ROC) curve analysis to differentiate patients with SLE from HC using relative circIBTK expression in the 42 patients with SLE and 35 HC (Fig. 1i). The area under the curve (AUC) was 0.728 and the 95% confidence interval (95% CI) was 0.610−0.845. This indicated that circIBTK might have good diagnostic value for SLE and could act as a potential diagnostic biomarker of SLE.

### CircIBTK served as a miRNA sponge for miR-29b

**Fig. 2:**
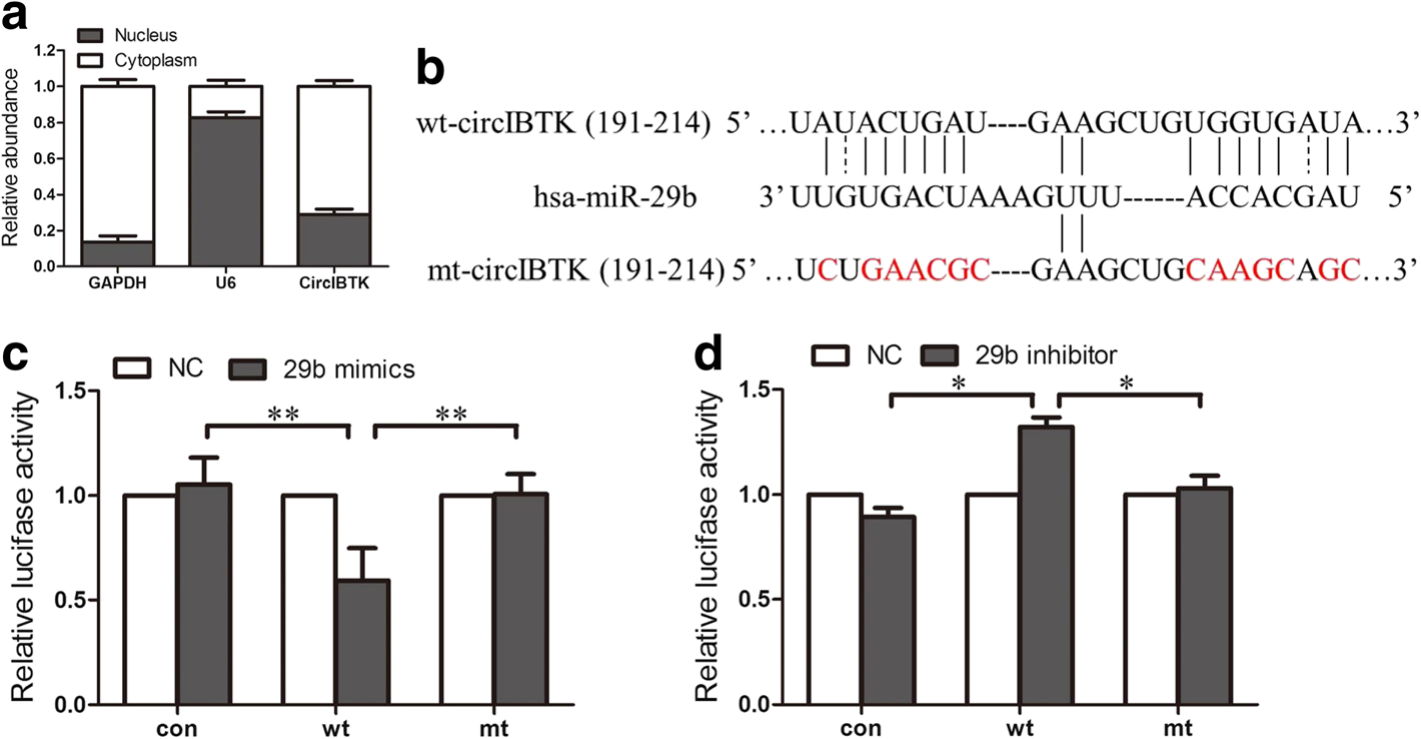
CircIBTK served as a miRNA sponge for miR-29b. **a** qRT-PCR analysis of nuclear and cytoplasmic RNAs showed that circIBTK was preferentially localized within the cytoplasm. GAPDH, glyceraldehyde-3-phosphate dehydrogenase. **b** Predicted binding sites and corresponding mutant sites of circIBTK and miR-29b (wt, wild-type; mut, mutant type). **c**, **d** Effects of miR-29b on the expression of luciferase reporter genes containing circIBTK wt/mt binding site. Luciferase activity was normalized to the value obtained in the cells transfected with NC oligonucleotides. Results were represented as mean ± SD (*n* = 3). *P < 0.05, **P < 0.01. Con, control

In order to explore the function of circIBTK in SLE, first the intracellular location of circIBTK in PBMCs was characterized. Nuclear and cytoplasmic fractions were separated from PBMCs and nuclear control transcript (U6) and cytoplasmic control transcript (GAPDH mRNA) were detected by qRT-PCR. This showed that circIBTK mostly existed in the cytoplasm of PBMCs (Fig. 2a). Then, we explored whether circIBTK could act as a miRNA sponge. Using software based on TargetScan and mi-Randa, miR-29b was found to have two potential binding sites on circIBTK (Fig. 2b). Then, we performed luciferase reporter assays to determine whether miR-29b could directly bind to circIBTK. We constructed a circIBTK fragment and inserted it immediately downstream of the luciferase reporter gene. MiR-29b mimic or miR-29 inhibitor was co-transfected with the luciferase reporters into PBMCs and resulting in miR-29b mimic reducing the luciferase reporter activity, while miR-29b inhibitor promoted the luciferase reporter activity. Next, we mutated the two miRNA target sites with the inclusion of the circIBTK sequence in the 3’ UTR. We found that miR-29b had no significant effect on luciferase activity (Fig. 2c and d). These results suggested that circIBTK might serve as a miRNA sponge for miR-29b.

### Mir-29b expression was upregulated in SLE and correlated with clinicopathological variables in patients with SLE

**Fig. 3:**
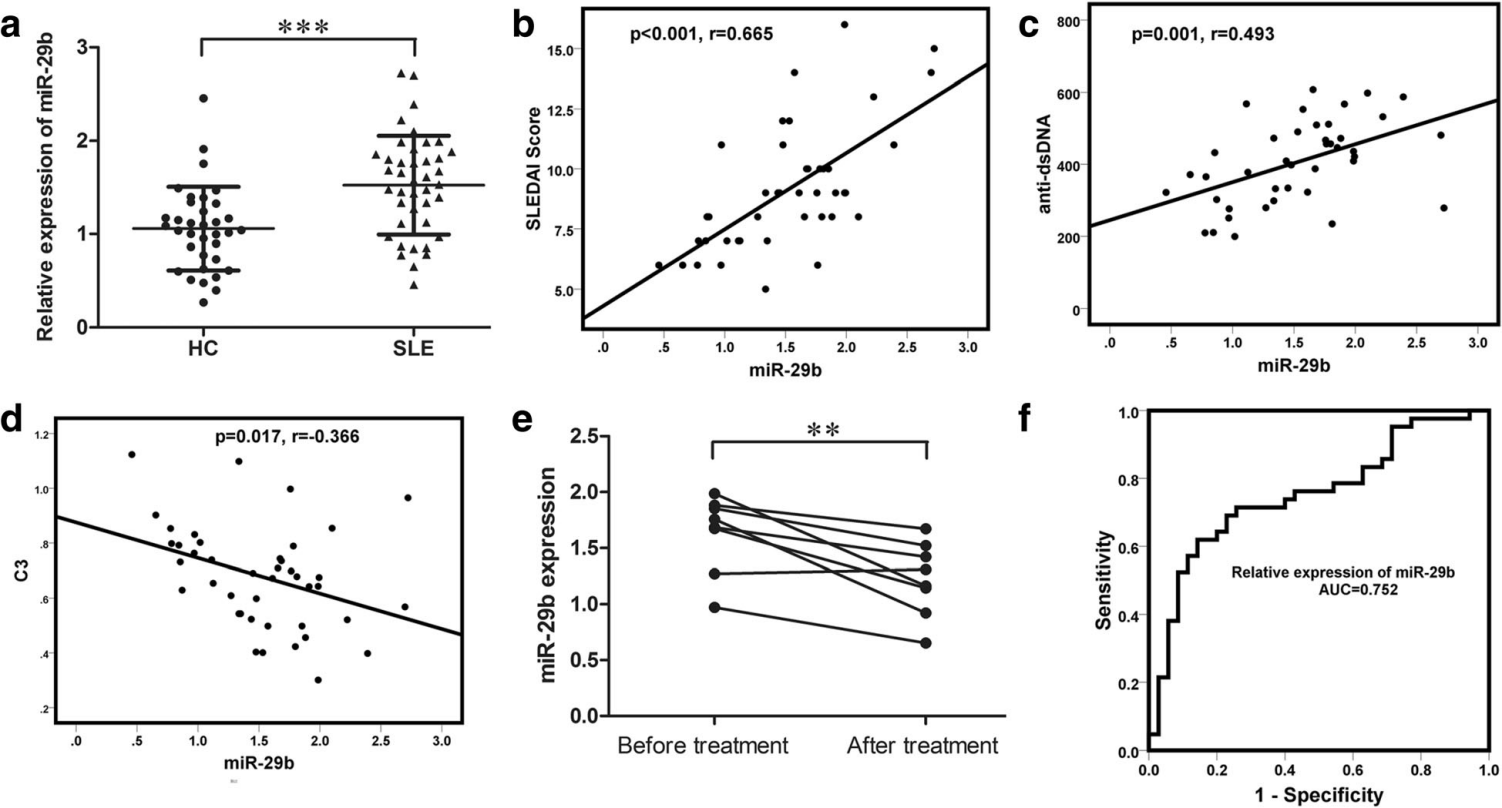
Mir-29b expression was upregulated in systemic lupus erythematosus (SLE) and correlated with clinicopathological variables in patients with SLE. **a** Expression of miR-29b in peripheral blood mononuclear cells (PBMCs) from 42 patients with SLE and 35 healthy controls (HC) compared using the unpaired Student’s *t* test. **b**-**d** Correlation between expression of circIBTK and Systemic Lupus Erythematosus Disease Activity Index (SLEDAI) score, anti-dsDNA titer or complement C3 level analyzed with Spearman’s analysis. **e** miR-29b expressions in eight patients who achieved significant clinical improvement after systematic treatment; data were compared using the paired Student’s *t* test. **f** ROC curve of relative miR-29b expressions for differentiating the 42 patients with SLE from 35 HC. Results are represented as mean ± SD. **P < 0.01, ***P < 0.001

In order to explore the function of miR-29b in SLE, we then detected the expression of miR-29b using qRT-PCR in PBMCs obtained from 42 patients with SLE and 35 healthy controls. The result showed that the expression of miR-29b was upregulated in SLE (Fig. 3a). Correlation between clinicopathological variables and miR-29b expression was tested to assess whether miR-29b might be a potential biomarker in the clinical estimate of the activity of SLE. There was strong positive correlation between miR-29b expression and the SLEDAI score in patients with SLE. Mir-29b expression was also positively correlated with anti-dsDNA titer and inversely correlated with complement C3 level (Fig. 3b, c and d). Accordingly, there also was significant decrease in miR-29b expression when eight patients achieved significant clinical improvement after systematic treatment (Fig. 1g and 3e). These results demonstrated that miR-29b could act as a biomarker to estimate the activity of SLE and verify the effectiveness of the treatment of SLE. To assess the diagnostic value of miR-29b in SLE, we also performed ROC curve analysis with the relative miR-29b expression in the 42 patients with SLE and 35 healthy controls (Fig. 3f). The AUC was 0.752 and the 95% CI was 0.642–0.862. This indicated that miR-29b might act as a potential diagnostic biomarker of SLE.

### CircIBTK could induce DNA methylation via miR-29b in SLE

**Fig. 4:**
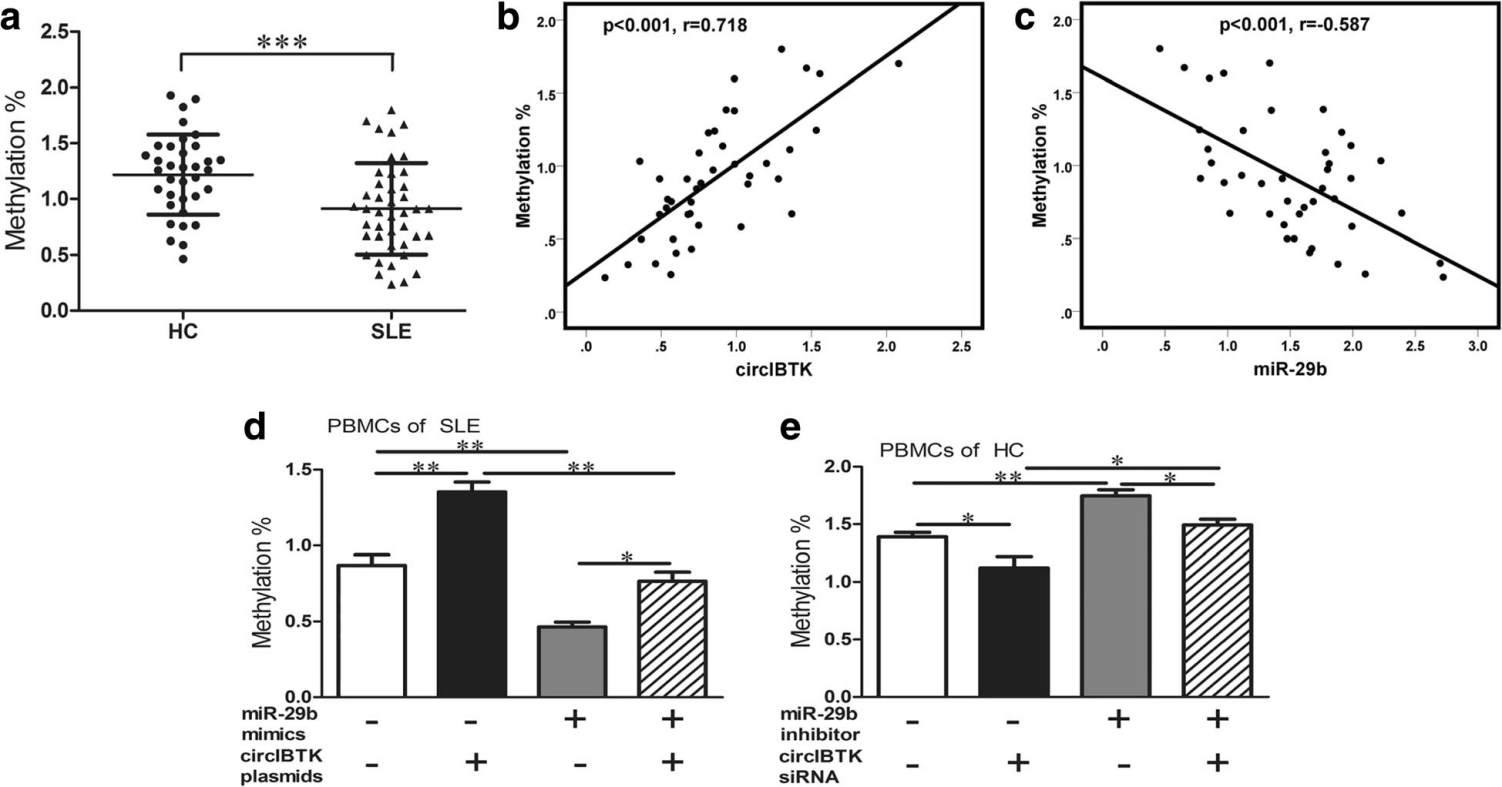
CircIBTK could induce DNA methylation via miR-29b in systemic lupus erythematosus (SLE). **a** Global DNA methylation in peripheral blood mononuclear cells (PBMCs) from 42 patients with SLE and 35 healthy controls (HC) compared using the unpaired Student’s *t* test. **b**, **c** Correlation between global DNA methylation and expression of circIBTK or miR-29b analyzed with Spearman’s analysis. **d** DNA methylation in PBMCs from patients with SLE, transfected with miR-29b mimics, circIBTK expression plasmids, NC oligonucleotides, or empty vector. **e** DNA methylation in PBMCs from HC, transfected with miR-29b inhibitor, circIBTK siRNA or NC oligonucleotides. Results are represented as mean ± SD (*n* = 3). *P < 0.05, **P < 0.01

To study the functions of circIBTK and miR-29b in the progression of SLE and explain why they can act as biomarkers of SLE, we further explored the mechanism. As miR-29b can induce DNA demethylation according to many studies [23–25], and DNA demethylation was common in patients with SLE [14, 15], we further explored whether DNA methylation in SLE could be regulated by circIBTK via miR-29b. We first measured global DNA methylation in PBMCs from patients with SLE and HC. DNA methylation was statistically lower in PBMCs from patients with SLE than that from healthy controls (Fig. 4a). We also tested correlation between DNA methylation level and circIBTK or miR-29b expression. The results showed that circIBTK expression was positively correlated with DNA methylation level and miR-29b expression was inversely correlated with DNA methylation level in SLE (Fig. 4b and c). To further explore the mechanism, PBMCs from patients with SLE were transfected with circIBTK expression plasmid or/and miR-29b mimics, and PBMCs from healthy controls were transfected with circIBTK siRNA or/and miR-29b inhibitor. DNA methylation levels were subsequently measured. The results showed that DNA methylation levels were increased when PBMCs were transfected with circIBTK expression plasmids or miR-29b inhibitor and decreased when PBMCs were transfected with circIBTK siRNA or miR-29b mimics (Fig. 4d and e). What is more, co-transfection of miR-29b mimics and circIBTK expression plasmids significantly attenuated the increasing effect on DNA methylation induced by circIBTK and the decreasing effect on DNA methylation induced by miR-29b. Co-transfection of miR-29b inhibitor and circIBTK siRNA reversed the decrease in DNA methylation induced by si-circIBTK and the increase of DNA methylation induced by miR-29b inhibitor (Fig. 4d and e). These results indicated that miR-29b could induce DNA demethylation and circIBTK could reverse miR-29b-induced DNA demethylation by regulating miR-29b in SLE.

### CircIBTK regulated the AKT signaling pathway by binding to miR-29b

**Fig. 5:**
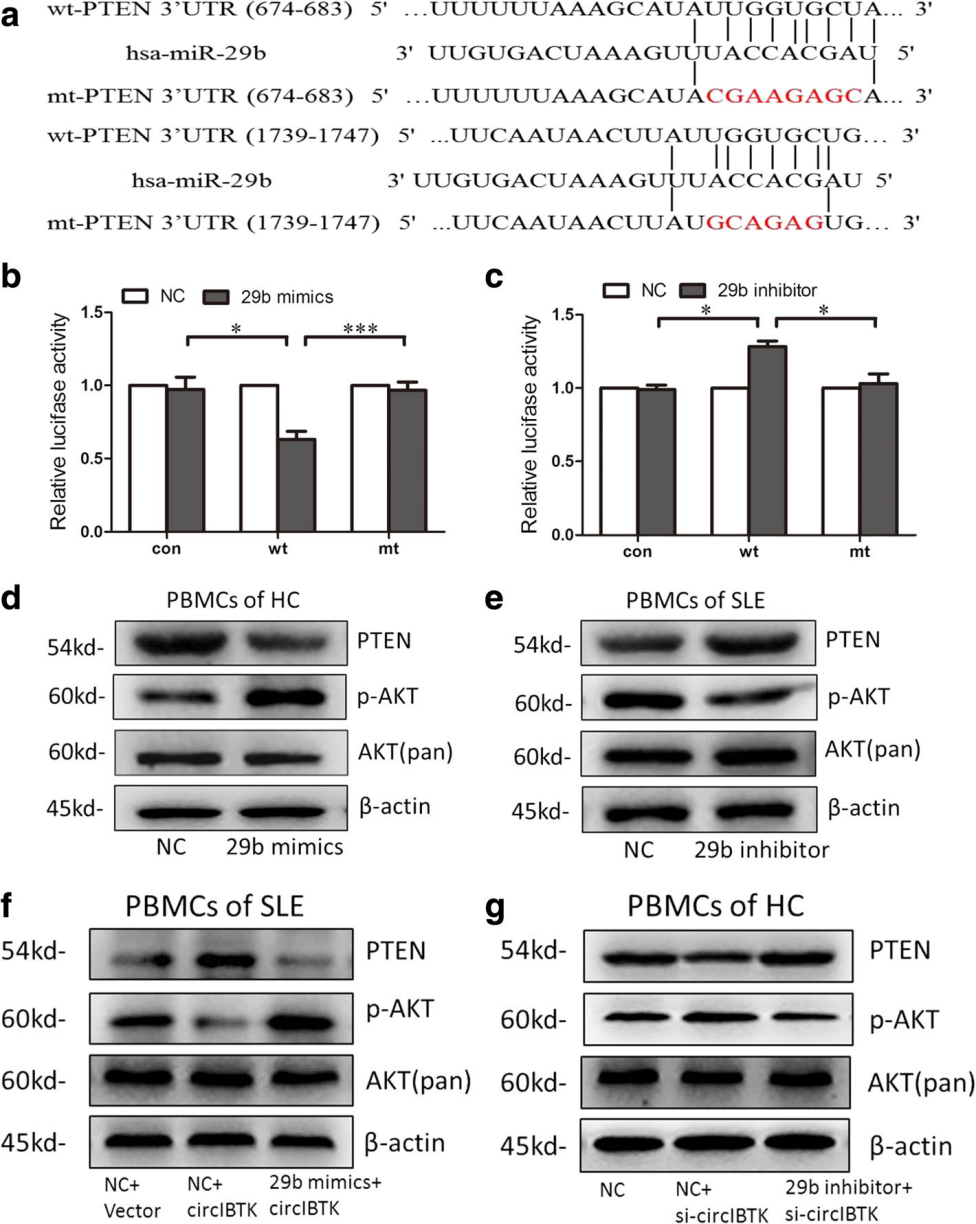
CircIBTK regulated the AKT signaling pathway by binding to miR-29b. **a** miR-29b predicted binding sites and corresponding mutant sites in the 3′ UTR of phosphatase and tensin homolog (PTEN) mRNA (wt, wild-type; mut, mutant type). **b**, **c** Effects of miR-29b on the expression of PTEN 3′ UTR-containing reporter genes. Luciferase activity was normalized to the value obtained in cells transfected with NC oligonucleotides. **d**, **e** Western blot analysis of PTEN expression and AKT phosphorylation in peripheral blood mononuclear cells (PBMCs) from healthy controls (HC), transfected with miR-29b mimics and PBMCs from patients with systemic lupus erythematosus (SLE), transfected with miR-29b inhibitor. **f** Western blot analysis of PTEN/AKT signaling-related proteins in PBMCs from patients with SLE, transfected with miR-29b mimics, circIBTK expression plasmids, NC oligonucleotides, or empty vector. **g** Western blot analysis of PTEN/AKT signaling-related proteins in PBMCs from HC, transfected with miR-29b inhibitor, circIBTK siRNA or NC oligonucleotides. Results are represented as mean ± SD (*n* = 3). *P < 0.05, ***P < 0.001

As the AKT signaling pathway may participate in the pathogenesis of SLE, we further explored whether it could be regulated by circIBTK via miR-29b. Through Targetscan and microRNA.org, we found PTEN might be a potential target of miR-29b (Fig. 5a). Then, we constructed reporter gene plasmids with PTEN 3’-UTR-containing wild-type (wt) or mutant (mut) miR-29b binding sites. Luciferase assay indicated that miR-29b mimics suppressed the expression of reporter gene carrying wt 3’UTR but not containing mut 3’UTR and miR-29b inhibitor promoted the expression of reporter gene carrying wt 3’UTR but not containing mut 3’ UTR (Fig. 5b and c). Furthermore, PTEN expression decreased while phosphorylation of AKT increased in PBMCs transfected with miR-29b mimics and PTEN expression increased while phosphorylation of AKT decreased in PBMCs transfected with miR-29b inhibitor (Fig. 5d, e and Additional file 2: Figure S1a-S1d). These data suggested that PTEN might be a target of miR-29b in PBMCs in SLE and miR-29b could activate the AKT signaling pathway by suppressing PTEN.

To explore whether circIBTK could regulate the PTEN/AKT signaling pathway via miR-29b, we transfected miR-29b mimics and circIBTK expression plasmids into PBMCs from patients with SLE. This showed that upregulated circIBTK promoted the expression of PTEN and inhibited the phosphorylation of AKT, and over expression of miR-29b significantly attenuated the circIBTK-induced increased expression of PTEN and deactivation of the AKT signaling pathway (Fig. 5f and Additional file 2: Figure S1e-S1f). Accordingly, we transfected miR-29b inhibitor and circIBTK small interfering RNA (siRNA) into PBMCs from HC. Downregulation of circIBTK resulted in decreased expression of PTEN and thereby increased phosphorylation of AKT and knockdown of miR-29b significantly reversed the PTEN downregulation and activation of the AKT signaling pathway induced by circIBTK inhibition (Fig. 5g and Additional file 2: Figure S1g-S1h). These data demonstrated that circIBTK could inhibit the activation of AKT signaling pathway in SLE by binding to miR-29b.

## Discussion

Recently, more and more research has focused on the function of circRNAs in many human diseases, while the expression profile and function of circRNAs in SLE remain unclear. In this study, we identified many circRNAs in SLE and found that some of these were differentially expressed in PBMCs from patients with SLE compared with healthy controls, which suggested that these RNAs might be regulated and exert a potential function.

CircIBTK, which was derived from the IBTK gene locus, was downregulated in patients with SLE. Bruton’s tyrosine kinase (BTK) is a member of the Tec family of non-receptor protein tyrosine kinases and a downstream signaling molecule of the B cell antigen receptor (BCR) signaling pathway, involved in the development, activation, and survival of B cells. Some studies have proved that BTK plays a significant role in the initiation and development of SLE. For example, transgenic mice over expressing BTK specifically in B cells produce antinuclear antibody and develop lupus-like symptoms [26]. Furthermore, when BTK inhibitor was used to treat the lupus mice, it alleviated damage in the kidney, and the production of autoantibody [27–29]. In humans, higher BTK expression in B cells from peripheral blood is associated with lupus nephritis [30]. Inhibitor of Bruton’s tyrosine kinase (IBTK) is an inhibitor of BTK that can bind to the PH domain of BTK to downregulate BTK kinase activity, BTK-mediated calcium mobilization, and nuclear factor kappa B-driven transcription [31]. We showed that the mRNA level of IBTK was downregulated in PBMCs from patients with SLE and this indicated that IBTK might be regulated or exert a potential function in SLE, which needed further exploration.

CircRNAs can act as potential biomarkers for disease and we showed that circIBTK is a marker of SLE. In this study, circIBTK was downregulated in SLE and the expression of circIBTK was strongly inversely correlated with the SLEDAI score. This revealed that the expression of circIBTK could help estimate the activity of SLE. Furthermore, circIBTK expression was notably increased when patients received efficacious treatment. So, circIBTK expression might indicate the effectiveness of treatment for SLE. What is more, the ROC curve also indicated that circIBTK might act as a potential diagnostic biomarker of SLE. We revealed the important role of circIBTK in SLE in this study.

In this study, we found that circIBTK might function as a miR-29b sponge. And we used luciferase reporter assays to verify this prediction. In patients with SLE compared to healthy controls, miR-29b levels were proved to be upregulated in CD4+ T cells [23]. We also proved that miR-29b levels were upregulated in PBMCs from patients with SLE compared to healthy controls. Like circIBTK, miR-29b also had the potential function of helping diagnose and estimate the activity and effectiveness of treatment of SLE. According to some studies, miR-29b could induce DNA demethylation via various ways [23–25]. Our results showed that miR-29b could induce DNA demethylation in SLE and miR-29b expression was inversely correlated with DNA methylation in SLE. What is more, expression of PTEN was decreased in SLE and the level of PTEN expression was inversely correlated with disease activity [32]. Anomalous activity of AKT kinases has been documented in peripheral blood B cells and T cells from patients with SLE [21, 22], and this may participate in the progress of SLE. We also proved that miR-29b could activate the AKT signaling pathway by suppressing PTEN expression by directly targeting PTEN. These results indicated that miR-29b could play a significant role in the pathogenesis of SLE, and might be a useful therapeutic target in SLE. Our data also demonstrated that circIBTK could inhibit DNA demethylation and activation of the AKT signaling pathway via the regulation of miR-29b in SLE. As DNA demethylation and activation of the AKT signaling pathway are critical for the initiation and development of SLE, and circIBTK and miR-29b could regulate both DNA demethylation and activation of the AKT signaling pathway, this explains why circIBTK and miR-29 correlated with clinicopathological variables in patients with SLE and act as biomarkers of SLE.

## Conclusions

In conclusion, circIBTK and miR-29b were abnormally expressed in PBMCs from patients with SLE and could regulate DNA methylation and activation of the AKT signaling pathway in PBMCs in SLE. Our study explained the important role of circIBTK and miR-29 in SLE progression and suggested that circIBTK and miR-29 could act as biomarkers and therapeutic targets for SLE.

## Additional files

Additional file 1: **Table S1.** Clinical characteristics of 42 patients with SLE and 35 healthy controls. (DOCX 16 kb)

Additional file 2: **Figure S1. a**, **b** Statistical analysis of the cumulative densitometry data for western blot analysis of PTEN expression and AKT phosphorylation in PBMCs from HC transfected with miR-29b mimics. **c**, **d** Statistical analysis of the cumulative densitometry data for western blot analysis of PTEN expression and AKT phosphorylation in PBMCs from patients with SLE, transfected with miR-29b inhibitor. **e**, **f** Statistical analysis of the cumulative densitometry data for western blot analysis of PTEN/AKT signaling-related proteins in PBMCs from patients with SLE, transfected with miR-29b mimics, circIBTK expression plasmids, NC oligonucleotides or empty vector. **g**, **h** Statistical analysis of the cumulative densitometry data for western blot analysis of PTEN/AKT signaling-related proteins in PBMCs from HC transfected with miR-29b inhibitor, circIBTK siRNA or NC oligonucleotides. Three replicate experiments were performed. The cumulative densitometry data were compared using the paired Student’s *t* test and results were represented as mean ± SD (*n* = 3). *P < 0.05, **P < 0.01. (PDF 298 kb)

## Abbreviations

95% CI: 95% Confidence interval
AUC: Area under the curve
BTK: Bruton’s tyrosine kinase
circRNA: Circular RNA
dsDNA: double-stranded DNA
GAPDH: Glyceraldehyde-3-phosphate dehydrogenase
HC: Healthy controls
IBTK: Inhibitor of Bruton’s tyrosine kinase
mRNA: Messenger RNA
miRNA: MicroRNA
ncRNA: Non-coding RNA
PBMC: Peripheral blood mononuclear cell
PTEN: Phosphatase and tensin homolog
qRT-PCR: Quantitative real-time PCR
ROC: Receiver operating characteristic
SLE: Systemic lupus erythematosus
SLEDAI: Systemic Lupus Erythematosus Disease Activity Index
UTR: Untranslated region
