# Supplementary material for: CircIBTK inhibits DNA demethylation and activation of AKT signaling pathway via miR-29b in peripheral blood mononuclear cells in systemic lupus erythematosus
Source: Arthritis Res Ther. 2018 Jun 8;20:118. doi: 10.1186/s13075-018-1618-8 (PMC5993996; doi:10.1186/s13075-018-1618-8)
Supplement: Supplementary file 1 — Table S1. Clinical characteristics of 42 patients with SLE and 35 healthy controls. (DOCX 16 kb) [file 13075_2018_1618_MOESM1_ESM.docx]

**Table S1.** Clinical characteristics of 42 SLE patients and 35 healthy controls.

| Factors | SLE(n=42) | HC(n=35) |
| --- | --- | --- |
| Gender(male/female) | 6/36 | 5/30 |
| Age (years)* | 32.12±7.17 | 31.15±6.33 |
| SLEDAI score* | 9.14±2.60 | NA |
| Anti-dsDNA (IU/ml) * | 405.47±113.63 | NA |
| C3 (g/l) * | 0.68±0.19 | NA |

*Data are mean ± SD.
