# Supplementary material for: CircIBTK inhibits DNA demethylation and activation of AKT signaling pathway via miR-29b in peripheral blood mononuclear cells in systemic lupus erythematosus
Source: Arthritis Res Ther. 2018 Jun 8;20:118. doi: 10.1186/s13075-018-1618-8 (PMC5993996; doi:10.1186/s13075-018-1618-8)
Supplement: Supplementary file 2 — Figure S1. a, b Statistical analysis of the cumulative densitometry data for western blot analysis of PTEN expression and AKT phosphorylation in PBMCs from HC transfected with miR-29b mimics. c, d Statistical analysis of the cumulative densitometry data for western blot analysis of PTEN expression and AKT phosphorylation in PBMCs from patients with SLE, transfected with miR-29b inhibitor. e, f Statistical analysis of the cumulative densitometry data for western blot analysis of PTEN/AKT signaling-related proteins in PBMCs from patients with SLE, transfected with miR-29b mimics, circIBTK expression plasmids, NC oligonucleotides or empty vector. g, h Statistical analysis of the cumulative densitometry data for western blot analysis of PTEN/AKT signaling-related proteins in PBMCs from HC transfected with miR-29b inhibitor, circIBTK siRNA or NC oligonucleotides. Three replicate experiments were performed. The cumulative densitometry data were compared using the paired Student’s t test and results were represented as mean ± SD (n = 3). *P < 0.05, **P < 0.01. (PDF 298 kb) [file 13075_2018_1618_MOESM2_ESM.pdf]

Figure S1

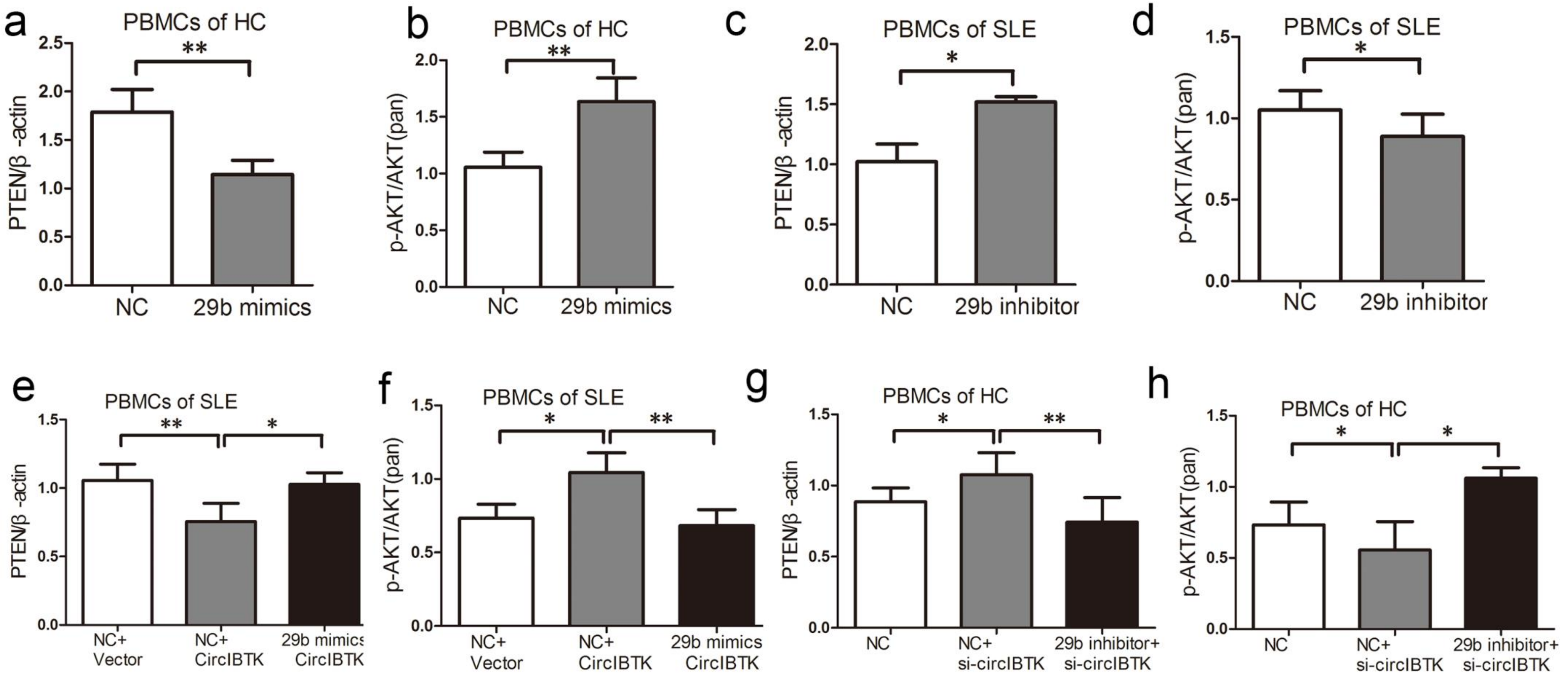

(a and b) Statistical analysis of the cumulative densitometry data for Western blot analysis of PTEN expression and AKT phosphorylation in PBMCs of HC transfected with miR-29b mimics. (c and d) Statistical analysis of the cumulative densitometry data for Western blot analysis of PTEN expression and AKT phosphorylation in PBMCs of SLE transfected with miR-29b inhibitor. (e and f) Statistical analysis of the cumulative densitometry data for Western blot analysis of PTEN/AKT signaling related proteins in PBMCs of SLE transfected with miR-29b mimics, circIBTK expression plasmids, NC oligonucleotides or empty vector. (g and h) Statistical analysis of the cumulative densitometry data for Western blot analysis of PTEN/AKT signaling related proteins in PBMCs of HC transfected with miR-29b inhibitor, circIBTK siRNA or NC oligonucleotides. Three replicate experiments were performed. The cumulative densitometry data were compared with paired Student's t-test and results were represented as mean± SD (n=3). *P<0.05, **P<0.01.
